# Children with computer game addiction have weakness in sustained attention

**DOI:** 10.1192/j.eurpsy.2021.1514

**Published:** 2021-08-13

**Authors:** N. Kiseleva, S. Kiselev

**Affiliations:** 1 Laboratory For Brain And Neurocognitive Development, Ural Federal University, Ekaterinburg, Russian Federation; 2 Clinical Psychology, Ural Federal University, Ekaterinburg, Russian Federation

**Keywords:** computer game addiction, sustained attention

## Abstract

**Introduction:**

Various digital technologies are increasingly being introduced into the everyday life of children. There are evidences that digital addiction has negative effect on cognitive functions of children. What kind of specific effect does this new “digital environment” have for children?

**Objectives:**

The goal of this research is to check the hypothesis that 7-year-old children with computer game addiction have weakness in sustained attention.

**Methods:**

We used questionnaire for parents to reveal children with computer game addiction. Experimental group consisted of 28 7-year-old children with computer game addiction. Control group consisted of 28 children without computer game addiction. Children from experimental and control group were matched for gender and IQ. To assess the sustained attention we used subtest from Luria’s child neuropsychological battery. This subtest is designed to assess visual sustained attention.

**Results:**

One-way ANOVAs by group revealed significant differences (p≤0,05) between the groups in the level of visual sustained attention.

**Conclusions:**

It can be assumed that computer game addiction has negative effect on the development of visual sustained attention in children. However, we need to do additional research to approve this preliminary results.

